# Characteristics and Outcomes of Patients With a Moderate History, ECG, Age, Risk Factors, and Troponin (HEART) Score

**DOI:** 10.7759/cureus.73343

**Published:** 2024-11-09

**Authors:** Leslie Jewett, Nicholas Hollman, Roger DesPrez, Andrew Baker, Emily McNulty, Joshua Gentges

**Affiliations:** 1 Emergency Medicine, CHI St. Vincent Hot Springs, Hot Springs, USA; 2 Biostatistics, The University of Oklahoma School of Community Medicine, Tulsa, USA; 3 Cardiology, Oklahoma Heart Institute, Tulsa, USA; 4 Emergency Medicine, Advocate Christ Medical Center, Oak Lawn, USA; 5 Emergency Medicine, Oklahoma State University Center for Health Sciences, Tulsa, USA; 6 Emergency Medicine, The University of Oklahoma School of Community Medicine, Tulsa, USA

**Keywords:** chest pain, cvd risk factors, emergency department disposition, heart score, mace, retrospective cohort, risk stratification

## Abstract

Introduction

Chest pain is a common, expensive cause of admission to the hospital from the Emergency Department (ED). The History, ECG, Age, Risk Factors, and Troponin (HEART) score is a risk stratification tool often used to determine the disposition of chest pain patients. This study evaluates the association of age, gender, HEART score, diabetes mellitus (DM), hypertension (HTN), hypercholesterolemia, family history (Fam Hx), and tobacco use with major adverse cardiovascular events (MACE) and hospital readmission. While low-risk HEART score patients are generally discharged and high-risk patients generally admitted, data is limited on the appropriate disposition strategy for moderate-risk patients. Our goal is to understand the risks of cardiovascular morbidity, the studies performed, and the factors associated with readmission in moderate HEART score patients.

Methods

A retrospective cohort study was conducted using ED records from March 1, 2018, to March 31, 2019. Patients with a moderate HEART score (4-6) were included. Data on additional testing and outcomes were collected. Statistical analyses included Pearson Chi-square tests and unadjusted and adjusted logistic regression to assess associations between explanatory variables and outcomes. Multicollinearity was assessed with all variance inflation factors below 1.3. To evaluate how well the logistic model performed an area under the curve (AUC) analysis was performed, with values of 0.68 (cardiovascular morbidity events) and 0.67 (readmission) respectively. No pre-existing AUC cutoff was used.

Results

The study included 959 patients with a moderate HEART score. The average age was 58.9 years, and 486 (50.7%) were male. Cardiovascular disease morbidity events occurred in 72 (7.5%) of patients, and 108 (11.3%) were readmitted within six weeks. Higher HEART scores, male gender, and diabetes were significantly associated with increased odds of cardiovascular morbidity. Male gender, tobacco use, and hypercholesterolemia were significantly associated with hospital readmission. The percutaneous intervention occurred in 60 (6.3%) patients, coronary bypass grafting in 10 (1.0%), and cerebrovascular accident in two (0.2%). Myocardial perfusion imaging was performed in 421 (43.9%) of patients and echocardiogram in 445 (46.4%), while computerized tomography coronary angiogram was performed in 99 (10.3%). No deaths were identified in our study.

Discussion

The findings suggest that higher HEART scores, male gender, and diabetes are significant predictors of cardiovascular morbidity, while male gender, tobacco use, and hypercholesterolemia predict hospital readmission. Reliance on ED records could limit study generalizability as we did not capture direct admissions. The retrospective design could introduce bias from confounding variables. Most of the testing and intervention during hospitalization could reasonably have been performed in an outpatient setting.

Conclusion

This study provides quantitative data on the utility of hospitalization for moderate-risk HEART score patients. The results support the potential for outpatient management in certain cases, especially those with HEART scores of 4. Future studies should include randomized controlled trials comparing the discharge of moderate HEART score patients with inpatient management.

## Introduction

Chest pain is the second most common complaint presented to Emergency Departments (EDs) and is the most expensive, costing hospitals in the largest database (29 million ED visits across 28 states) in the U.S. an average of $18,000 per admission [1]. The History, ECG, Age, Risk Factors, and Troponin (HEART) score is a risk stratification tool utilized in the ED to aid in the determination of appropriate dispositions for patients presenting with chest pain and possible acute coronary syndrome [2]. The score sorts patients into low, medium, and high risk based on history, ECG findings, age, risk factors, and troponins [2]. In the original study, for those with a low-risk score of 0-3, the risk of major adverse cardiac events (MACE, defined in this study as acute myocardial infarction, coronary bypass grafting, percutaneous intervention, and death) was 2.5%, patients scoring 4-6 had MACE at 20.3%, and patients scoring 7-10 had MACE at a rate of 72.7%. In that study, low-risk patients were discharged, moderate-risk patients were admitted, and high-risk patients were referred for rapid intervention [2]. Multicenter and multinational validation studies have been performed and found lower rates than the original paper [3-5]. For example, in a multicenter validation study by the same study group that performed the initial study rates of MACE were 0.99%, 11.6%, and 65.2% for low, moderate, and high risk, respectively [4]. In a large, multicenter U.S. validation study, rates were much lower at 0.2%, 1.0%, and 3.0% for the respective risk categories, and no overlap in the confidence intervals [5]. It is unclear why U.S. rates were lower than those found in other studies; the U.S. study did not give participant characteristics or breakdowns by HEART score components, but did have a higher percentage (59%) of patients in the low-risk category.

The caution with which exclusive use of the HEART score should be exercised has been addressed by Webb et al. with the patients who are placed in the moderate risk category based solely on age and risk factors [6]. There are also subjective measures in the HEART score, causing variations between clinicians’ scores. If used in isolation, this would subjectively alter disposition decisions [7]. The most subjective components are the history and ECG interpretation. There is evidence suggesting the highest predictive value for MACE in the HEART score is the history and troponin level [8]. This is concerning because history, a strong predictor of MACE, is also the component of the HEART score with the lowest interrater reliability, which may lead to unnecessary hospital admission in some cases. Subjectivity in the HEART score might also lead to inappropriate discharge if patients are placed in too low of a risk category. In the moderate-risk group, discharge with expedited follow-up may be appropriate, and many studies have attempted to address this [8-11], but randomized trials are lacking and the studies are not standardized. Sharp et al. show a 10% rate of unnecessary hospital admissions for chest pain patients, raising a concern that the subjectivity of the HEART score may drive some of these admissions [10].

Multiple studies have demonstrated no increase in mortality from discharging moderate-risk HEART score patients [8,9,11]. In Nevada, a study of 206 patients referred to a cardiology clinic in an expedited manner five had a MACE with no deaths due to delays in care [8], although follow-up was not addressed. In a Canadian study where moderate HEART score patients were referred to urgent outpatient follow-up, MACE at six weeks was 9.3%, with only 1.1% occurring before the rapid access clinic appointment [9]. Coronary angiography was their highest MACE event, and with it excluded, MACE before the clinic appointment dropped to 0.7% [9]. The 0.7% is more clinically relevant as it includes parameters of acute myocardial infarction, revascularization, and death which is most consistent with other studies evaluated. Interestingly, the rate of low-risk patients in this study was similar (58%) to that found in the U.S. validation study [5].

In Sharp et al. and Barbarash et al., moderate HEART score patients were discharged with expedited cardiology follow-up, and no deaths were reported [8,10]. Similarly, Moustapha et al. found no difference in outcome between hospitalized and discharged patients presenting to the ER with chest pain [9]. This was with the hospitalized cohort having increased comorbidities and troponin levels. One of the limitations discussed in this paper was the types of diagnostic tests or interventions associated with hospitalization. Our paper addresses this issue by evaluating the "difficult" category of moderate risk HEART score patients, by testing what risk factors predict cardiovascular disease (CVD) morbidity events or hospital readmission among these patients, and by testing if the prevalence of hospital interventions differs by HEART score. Our goal is to understand the moderate HEART score for patients better and use that information to help clinicians make informed decisions in this group, including the critical question of disposition - hospitalization or outpatient care?

## Materials and methods

Study design

We used a retrospective cohort design for this study. ED records were reviewed for components of HEART score and then reviewed for additional testing and interventions related to chest pain (coronary computed tomography angiography (CCTA), echocardiogram (TEE), cardiac catheterization (CATH), percutaneous coronary intervention (PCI), coronary artery bypass grafting (CABG)) completed in the following 48-72 hours outside of the ED, but during the hospitalization. The HEART score was calculated by the reviewer using the categories of history, age, ECG findings, risk factors, and troponin, then further chart review for those with a moderate risk score was performed with the presented data being collected. Spot checks were performed on random patients to assess interrater reliability. If the ED record documented a HEART score, an independent calculation was made by the reviewer with the exception of deferring to the clinician's judgment of the relevance of the history. A convenience sample of patients admitted to the hospital for chest pain over a one-year period, from March 1, 2018, to March 31, 2019, was used for the analysis to attempt to avoid potential confounding by reducing non-covid admissions during the COVID-19 pandemic [12].

Statistical analysis

The explanatory variables of interest in the analysis were age (years), gender (male or female), hypertension (HTN), diabetes, hypercholesterolemia, family history of coronary artery disease (CAD), tobacco use, and HEART score. The sample was restricted to patients with a moderate HEART score of 4-6. The primary outcome was the presence of a CVD morbidity event (as no mortality events occurred during the time specified), including PCI, CABG, heart failure, and cerebrovascular accident (CVA). We intentionally did not code this as MACE since there is no standardization across studies as to what constitutes a MACE and we wanted to capture heart failure and CVA events which are sometimes but not always included in MACE. These were all equally weighted so the presence of any of these factors resulted in a positive cardiovascular morbidity event. No other outcomes were considered in defining the cardiovascular morbidity event outcome The secondary outcome was readmission for chest pain (as listed in the chart as the reason for readmission) within six weeks.

Distributions, frequencies, and appropriate assumptions were checked prior to all analyses. For descriptive statistics, data is displayed as percentages and frequencies for categorical variables and mean and standard deviation for continuous variables. Missing data was handled by listwise deletion, with missing data excluded on analyses with observations missing data. The assumption of independence was met by each observation representing a unique patient in the dataset. The assumption of linearity was needed only for age, tested by plotting this continuous predictor by the log odds of the predicted variables, with no violation seen. Pearson Chi-square tests, unadjusted logistic regression, and adjusted logistic regression analysis were performed to assess the association between the explanatory variables of interest and the outcomes defined. For the multivariable model, all variables found to be significant at a cut-off of p < 0.20 at the univariate level were entered into the model. This cutoff was used to capture all possible predictors related to our outcomes in the model potentially related to our outcomes. We then report the final significance of factors remaining significant at p < 0.05 in the final model. Multicollinearity in the final models was assessed using the variance inflation factor (VIF), with no evidence of multicollinearity. Previous literature [13] has shown no significant interactions between HEART score variables, and our initial model testing confirmed a lack of interaction. VIFs for each predictor in the CVD morbidity event model were gender=1.007, diabetes=1.049, tobacco use=1.011, HEART score=1.047. For the readmission model, VIF was as follows: gender=1.013, HTN=1.115, hypercholesterolemia=1.249, family history=1.057, tobacco use=1.018, HEART score=1.099. The strength of association in the adjusted logistic regression model is displayed as adjusted odds ratios (aOR) and 95% confidence intervals (CI). Model performance was measured using the area under the curve (AUC), without predefined cutoffs since there is no universally accepted acceptable performance. AUC values were 0.68 for the morbidity event model and 0.67 for the readmission model. All analyses were performed with a significance level of α=0.05. Data analysis was performed using the Statistical Analysis System (SAS) (version 9.4; SAS Institute Inc., Cary, NC). The study was approved by the Hillcrest Medical Center Institutional Review Board using a reliance agreement with the University of Oklahoma Health Sciences Center under approval number 21-08A.

## Results

There were 959 unduplicated patients over the one-year period from chart review with a HEART score of 4-6. Interrater reliability was evaluated by a random check of duplicated scores, with 66 exact matches out of 103 samples (64.08%) and a linearly weighted kappa of 0.440. Of the sample, 486 participants (50.7%) were male, with an average age of 58.9 years (SD = 12.0). In our sample, 72 (7.5%) experienced a CVD morbidity event, with 60 (6.3%) of these being percutaneous intervention and 10 (1.0%) being CABG. Two patients (0.2%) had a CVA, and no patients with CABG or CVA had PCI performed. A total of 108 (11.3%) were readmitted to the hospital for chest pain within six weeks. Descriptive statistics of the explanatory variables of interest and outcomes are provided in Table 1.

**Table 1:** Characteristics of the study cohort (n=959) ^a^Percutaneous coronary intervention; ^b^Coronary artery bypass grafting; ^c^Cerebrovascular accident; ​​​​​​​^d^Myocardial perfusion scan; ​​​​​​​^e^Transesophageal echocardiogram; ​​​​​​​^f^Cardiac catheterization; ​​​​​​​^g^Computerized tomography coronary angiogram; CAD: Coronary artery disease

Variable	Frequency (%)
Age (n=947) mean (SD)	58.9 (12.0)
HEART Score mean (SD)	5.0 (0.8)
4	281 (29.3)
5	361 (37.6)
6	317 (33.1)
Gender	
Male	486 (50.7)
Female	473 (49.3)
Tobacco Use (n=957)	331 (34.6)
Family History of CAD (n=957)	291 (30.4)
Diabetes (n=957)	361 (37.7)
Hypertension (n=957)	783 (81.8)
Hypercholesterolemia (n=957)	638 (66.7)
Cardiovascular Disease Morbidity Event	72 (7.5)
Readmission (n=955)	108 (11.3)
PCI^a^	60 (6.3)
CABG^b^	10 (1.0)
CVA^c^	2 (0.2)
Heart Failure	0 (0.0)
Death	0 (0.0)
MPS^d^	421 (43.9)
No test done	227 (23.7)
Other testing	4 (0.4)
TEE^e^	445 (46.4)
CATH^f^	111 (11.6)
CT^g^	99 (10.3)
Pacemaker	2 (0.2)

When analyzing study variables associated with a CVD morbidity event at the univariate level, continuous HEART score, gender, and diabetes were significantly associated at p < 0.05. An additional factor to be included in the multivariable model was tobacco use, though it was slightly above the cut-off of p < 0.20. In addition, variables significantly associated with hospital readmission at the univariate level were gender, tobacco use, HTN, and hypercholesterolemia. Continuous HEART score, family history of CAD, and diabetes status were additional factors that met the cut-off of p < 0.20 to be included in the multivariable model. Specifics on the prevalence of each outcome by exposures of interest can be seen in Table 2.

**Table 2:** Prevalence and unadjusted association between variables of interest and cardiovascular disease morbidity event and readmission *Statistically significant difference at p < 0.05, the prevalence of the outcome (cardiovascular disease morbidity event, readmission) is significantly different between groups for the variable of interest (gender, tobacco, etc.) ** Cochran-Armitage trend testing shows p = 0.0936 CAD: Coronary artery disease

Variable	Cardiovascular Event Frequency (%)	P-value	Readmission Frequency (%)	P-value 2
Age	-	0.852	-	0.403
HEART Score	-	< 0.001*	-	0.101**
4	11 (3.9)	-	23 (8.2)	-
5	24 (6.7)	-	45 (12.5)	-
6	37 (11.7)	-	40 (12.6)	-
Gender	-	0.005*	-	<0.001*
Male	48 (9.9)	-	71 (14.7)	-
Female	24 (5.1)	-	37 (7.9)	-
Tobacco Use	-	0.207	-	0.035*
Yes	20 (6.0)	-	47 (14.3)	-
No	52 (8.3)	-	61 (9.7)	-
Family History of CAD	-	0.408	-	0.073
Yes	25 (8.6)	-	41 (14.1)	-
No	47 (7.1)	-	67 (10.1)	-
Diabetes	-	0.006*	-	0.053
Yes	38 (10.5)	-	50 (13.9)	-
No	34 (5.7)	-	58 (9.8)	-
Hypertension	-	0.507	-	0.042*
Yes	61 (7.8)	-	96 (12.3)	-
No	11 (6.3)	-	12 (6.9)	-
Hypercholesterolemia	-	0.435	-	<0.001*
Yes	51 (8.0)	-	90 (14.1)	-
No	21 (6.6)	-	18 (5.7)	-

After adjusting for other factors included in the multivariable model (e.g. gender, tobacco use, diabetes), those with higher HEART scores had higher odds of a CVD morbidity event (aOR 1.65, 95% CI 1.18 to 2.31), those that identified as the male had higher odds of a CVD morbidity event (aOR 2.05, 95% CI 1.23 to 3.42), and people with diabetes had higher odds of a CVD morbidity event (aOR 1.66, 95% CI 1.01 to 2.73) (Table 3). With the same adjustments in the readmission model, those who identified as male gender (aOR 2.02, 95% CI 1.32 to 3.10), used tobacco (aOR 1.70, 95% CI 1.12 to 2.59), and those with high cholesterol (aOR 2.47, 95% CI 1.39, 4.40) had higher odds of hospital readmission. The AUC for the CVD morbidity event model and readmission model was 0.68 and 0.67, respectively.

**Table 3:** Multivariable analysis for odds of a cardiovascular disease morbidity event and odds of hospital readmission ^a^aOR: Adjusted odds ratio; CAD: Coronary artery disease *Statistically significant at p < 0.05, the beta coefficient in the logistic regression model is significantly different than zero.

Event Category	aOR^a^ (95% CI)		P-value
Cardiovascular disease morbidity event			
HEART Score (each unit increase)		1.65 (1.18, 2.31)	0.004*
Gender (M v F)		2.05 (1.23, 3.42)	0.006*
Tobacco Use		0.74 (0.43, 1.28)	0.282
Diabetes		1.66 (1.01, 2.73)	0.048*
Hospital Readmission			
HEART score (each unit increase)		0.98 (0.74, 1.30)	0.893
Gender (M v F)		2.02 (1.32, 3.10)	0.001*
Tobacco		1.70 (1.12, 2.59)	0.013*
Family History of CAD		1.28 (0.83, 1.98)	0.263
Diabetes		1.29 (0.84, 1.97)	0.244
Hypertension		1.32 (0.68, 2.56)	0.416
Hypercholesterolemia		2.47 (1.39, 4.40)	0.002*

Subgroup analysis (interventions by HEART score)

The prevalence of each individual outcome used to create our composite outcomes is summarized in Table 4. Patients with higher HEART scores trended towards a higher prevalence of hospital interventions. For the entire sample, the interventions/outcomes with the highest prevalence were readmission (108 (11.3%)), PCI (60 (6.3%)), MPS (421 (43.9%)), TEE (445 (46.4%)), CATH (111 (11.6%)), and CT (99 (10.3%)). Chi-square tests were then performed to determine if these interventions/outcomes significantly differed by HEART score. As seen in Table 4, the prevalence of PCI (p= 0.002), TEE (p=0.004), and CATH (p < 0.001) significantly differed by HEART score. The highest percentage of PCI occurred among those with a HEART score=6 (32 (10.1%)) compared to those with a HEART score=5 (19 (5.3%)), followed by the lowest PCI prevalence among those with a HEART score=4 (9 (3.2%)). For TEE, there was a higher prevalence among patients with a HEART score=6 (170 (53.6%)) compared to 161 (44.6%) among patients with a HEART score=5 and 114 (40.6%) among those with a HEART score=4. Finally, the highest prevalence of a CATH test was among those with HEART score=6 (54 (17.0%)) compared to those with HEART score=5 (41 (11.4%)) and HEART score=4 (16 (5.7%)). Though not statistically significant (p=0.158), there is a trend of higher readmission amongst those with a HEART score of 5 (45 (12.5%)) and 6 (40 (12.6%)), compared to those with a HEART score of 4 (23 (8.2%)).

**Table 4:** Prevalence of different interventions stratified by HEART score (n=959) *Statistically significant difference at p < 0.05, the prevalence of the intervention is significantly different between HEART score groups. -** Expected frequency too low to meet Chi-square test assumptions, N too high for Fisher's exact test, no inferential statistics performed to test for significant differences between HEART score groups. ^a^Percutaneous coronary intervention; ^b^Coronary artery bypass grafting; ​​​​​​​^c^Cerebrovascular accident; ​​​​​​​^d^Myocardial perfusion scan; ​​​​​​​^e^Transesophageal echocardiogram; ​​​​​​​^f^Cardiac catheterization; ​​​​​​​^g^Computerized tomography coronary angiogram

Intervention	Total sample - Frequency (%)	HEART Score=4 (n=281) - Frequency (%)	HEART score=5 (n=361) - Frequency (%)	HEART score=6 (n=317) - Frequency (%)	P-value
Readmission	108 (11.3)	23 (8.2)	45 (12.5)	40 (12.6)	0.158
PCI^a^	60 (6.3)	9 (3.2)	19 (5.3)	32 (10.1)	0.002*
CABG^b^	10 (1.0)	1 (0.4)	4 (1.1)	5 (1.6)	-**
CVA^c^	2 (0.2)	1 (0.4)	1 (0.3)	0 (0.0)	-**
Heart Failure	0 (0.0)	0 (0.0)	0 (0.0)	0 (0.0)	-**
Death	0 (0.0)	0 (0.0)	0 (0.0)	0 (0.0)	-**
MPS^d^	421 (43.9)	114 (40.6)	165 (45.7)	142 (44.8)	0.397
TEE^e^	445 (46.4)	114 (40.6)	161 (44.6)	170 (53.6)	0.004*
CATH^f^	111 (11.6)	16 (5.7)	41 (11.4)	54 (17.0)	< 0.001*
CT^g^	99 (10.3)	39 (13.9)	34 (9.4)	26 (8.2)	0.058
Pacemaker	2 (0.2)	1 (0.4)	0 (0.0)	1 (0.3)	-**
Other	4 (0.4)	2 (0.7)	1 (0.3)	1 (0.3)	-**

The results of the CATH tests are presented in Table 5. Patients with CATH results other than the three categories described were excluded (n=5). CAD presented with no intervention to follow and patients having an intervention were split as the most common result (74 (42.8%)). The least common result was an interpretation of no CAD present (25 (14.5%)). Although there was not a statistically significant association between HEART score and CATH results (p=0.492), the highest prevalence of interventions was seen amongst participants with a HEART score of six (43 (49.4%)).

**Table 5:** CATH results overall and by HEART score (n=173) ^a^Coronary artery disease; ^b^Percutaneous coronary intervention; ​​​​​​​^c^Coronary artery bypass grafting; CATH: Cardiac catheterization

Total sample - Frequency (%)		HEART Score=4 (n=25) - Frequency (%)	HEART score=5 (n=61) - Frequency (%)	HEART score=6 (n=87) - Frequency (%)	P-value
No CAD^a^	25 (14.5)	4 (16.0)	10 (16.4)	11 (12.6)	0.492
CAD^a^ present no intervention	74 (42.8)	11 (44.0)	30 (49.2)	33 (37.9)	
Intervention (Angioplasty, PCI^b^, CABG^c^, Angioplasty + PCI^b^)	74 (42.8)	10 (40.0)	21 (34.4)	43 (49.4)	

## Discussion

The CVD morbidity events calculated for our cohort were between the percentages for MACE reported by Backus’ multicenter validation study of the HEART score and Sharp’s United States validation study [4,5]. This may be because of differences in patient demographics, or because our analyses concentrated only on moderate-risk HEART patients. None of the adverse events included death in this cohort. Traditionally "high-risk" clinical factors such as HTN (783 (81.8%)) and hypercholesterolemia (638 (66.7%)) were very common in our cohort, likely due to the high regional prevalence of these clinical factors. These variables, however, did not predict cardiovascular risk. Hypercholesterolemia was predictive of readmission, perhaps due to clinicians rating it highly as a risk factor. The most common inpatient tests were TEE (445 (46.4%)), MPS (421 (43.9%)), CATH (111 (11.6%)), cardiac CT (99 (10.3%)) and PCI (60 (6.3%)). It is possible that the increased rate of intervention by HEART score in this study is because of clinician expectation of increased CVD by HEART score, but less clear is the benefit to patients of performing these interventions during a hospital stay. Seven in 10 patients had additional cardiac testing done while in the hospital, but multiple studies suggest outpatient management as a viable option with no increases in mortality [8,9]. The diagnostic tests performed do not inherently need to be completed in the hospital, and precedence has already been established for outpatient management [8,14,15]. No mortality was seen in our patient population, consistent with other studies showing no [8,9] or less than 1% [5,11] death rates in moderate HEART score patients.

For each of the tests or interventions, the rates increased with increasing HEART score, which is clinically consistent with increasing risk of a CVD morbidity event and consistent with previous reporting. Each unit increase in the HEART score represented a 65% increase in morbidity event risk, with a confidence interval between 18% and 131%. In our model, male gender and diabetes were most predictive of cardiovascular risk, while gender, diabetes, and tobacco use were all predictive of readmission. Tobacco use was not predictive of cardiovascular morbidity in our model when adjusted for other variables, perhaps due to sample size, correlation, or unmeasured confounders. The HEART score was not predictive of readmission, understandable since it is designed to evaluate cardiovascular risk rather than readmission. For the patients with a score of 4, a heart catheterization was performed in 16 patients (5.7%). This low rate could support the growing trend of outpatient discharge for these patients [8-10]. While readmission occurred in only 23 (8.2%) of these patients, PCI or CABG was performed on 10 the 281 (2.8%), though as stated previously, this does not exclude the potential for outpatient management, it does support the need for expeditious follow up [8,9,11,14,15]. For patients who received CATH, there was no statistically significant difference between rates of CAD regardless of HEART score. This retrospective study cannot show causation, but plausible mechanisms include clinician gestalt, errors in HEART score recording, and similar underlying pathology in moderate HEART score patients presenting with chest pain. In the multivariable model, higher odds of a CVD morbidity event were identified with increasing HEART scores in males and in diabetics. This is consistent with a multicenter validation study performed which identified these factors as independent risk factors for MACE [5]. While not part of the standard evaluation for the cohort studied here, cardiac CT angiography has become a potentially effective and cost-efficient option for the evaluation of these patients [16-18]. For patients with suspected CAD, there is a sensitivity of 85-99% and specificity of 64-92% [17], and this type of testing may lead to fewer admissions. This low specificity may lead to the unintentional consequence of increased invasive testing, as seen with D-dimer use [19]. This is why Yu et al. found that cardiac CT was best used as part of an algorithm that incorporates CT with myocardial perfusion imaging as opposed to being used in isolation [17]. Further study of cardiac CT angiography in moderate-risk patients is necessary.

Limitations

The primary endpoint of CVD morbidity event being used instead of MACE limits the direct comparison to other studies, but MACE is poorly defined [20] and clinicians should be cautious when comparing studies using MACE as an endpoint. This single-center study could be biased by loss to follow-up; patients who were discharged and subsequently were admitted to another facility might have different characteristics from those who stayed in the system. This was mitigated by the EMR being used by the three large hospital systems in the metropolis area as well as smaller sites in the surrounding rural areas. A limitation, though, is that patients might move outside of our area and be lost to follow-up. The cohort of patients was from an urban ED in a single city and is not necessarily generalizable to all patients presenting with chest pain. Variations in risk factors (for example, higher levels of HTN or lower levels of diabetes) should be considered by clinicians when evaluating the study's external validity. Convenience sampling could bias the results by seasonal variation. Local cardiology practice standards may differ from other localities, and it follows that not all systems may perform the same interventions on initial hospitalization; this would lead to changes in readmission patterns. Unmeasured confounders or reliance only on ED records could also limit generalizability.

## Conclusions

This retrospective analysis of interventions performed in the hospital for patients with moderate-risk HEART scores provides additional quantitative data regarding the utility of hospitalization for this demographic of patients by evaluating the interventions done during the hospitalization. The majority of interventions performed were not what can be considered emergent (TEE, MPS, CT), which supports the concept that not all moderate-risk HEART score patients require hospitalization. Since CCTA is highly sensitive for identifying CAD, clinical guidelines for managing these patients should incorporate this test and could reduce inpatient admissions, which would save hospital resources and reduce low-yield testing. Using CCTA could reduce the number of interventions, especially heart catheterization. Further study areas should include more outpatient testing and follow-up for these patients as well as studies on shared decision-making with patients regarding the need for hospitalization following presentation to the ER with chest pain. A multi-center, randomized trial comparing outcomes in moderate HEART patients who are discharged versus those who are hospitalized would help answer this question. While this retrospective study cannot demonstrate causal associations, it provides more evidence that many moderate HEART score patients, especially those with HEART scores of 4 can be managed with urgent outpatient follow-up. Male patients or those with diabetes were at increased risk in our group, and clinicians should consider those risk factors when making disposition decisions.
